# Improved protection of filtering facepiece through inactivation of pathogens by hypertonic salt solutions – A possible COVID-19 prevention device

**DOI:** 10.1016/j.pmedr.2020.101270

**Published:** 2020-11-28

**Authors:** Franz Tatzber, Ulrike Resch, Meinrad Lindschinger, Gerhard Cvirn, Willibald Wonisch

**Affiliations:** a Otto Loewi Research Center, Division of Immunology and Pathophysiology, Medical University of Graz, Austria; b Department of Vascular Biology and Thrombosis Research, Medical University of Vienna, Austria; c Institute of Nutritional and Metabolic Diseases, Outpatient Clinic Laßnitzhöhe, Austria; d Otto Loewi Research Center, Division of Physiological Chemistry, Medical University of Graz, Austria

**Keywords:** Filtering facepiece, Hypertonic saline, COVID-19, Protection

## Abstract

The filtering facepiece operates through filtration without the ability to kill the viruses. If the filtration might be combined with antiviral agents simultaneously in the masks, this would be much more efficient during the use of these masks and against cross-infection after being discarded. For centuries, sodium chloride (NaCl) contributes to inhibiting pathogens on various occasions. If aerosol with infectious agents reaches the filtering face-piecé surface of the filtering face-piece, coated with hypertonic saline, they become attracted by hygroscopic salt crystals. Proteins and nucleic acids lose their structural integrity and become inactivated concerning their infectious properties. We provide further evidence for cell growth inhibition with hypertonic saline in yeast cells comprising a defending cell wall. Proliferation was inhibited in a concentration-dependent manner, i.e., above 50 g/L, yeast cell proliferation was completely blocked. At a NaCl concentration of 100 g/L, even decomposition of the original inoculated organisms was observed. Therefore, we conclude that hypertonic saline- coated filtering facepiece might strongly reduce the numbers of infectious particles on their surfaces and thus protect mask carriers efficiently from infections.

## 1 Introduction

For centuries it is well known that sodium chloride contributes to germ inhibition on various occasions. For example, the Dead Sea and salt lakes contain a high sodium chloride concentration with practically no microorganisms (Williams, 1993). The hypertonic environment outside the cell is responsible for water diffusion from the cell, associated with dehydration and shrinking of the cell. This is the reason why nutritional meat gained durability if salted extensively.

A key task of hypertonic saline solution is widespread and rapid protein damage due to aggregation and misfolding of diverse proteins and water loss (Burkewitz et al., 2011). Not only proteins are affected by denaturation, but also nucleic acids. Virus particles consist predominantly of nucleic acids and proteins and may be especially sensitive to hypertonic saline. If denatured by salt, it is assumable that their surface proteins become destructured and their binding capacity to cell receptors is at least strongly reduced if not inhibited.

Lungs are marked with a large surface exposed to the external environment. Aerosolized hypertonic saline solution was suggested to reduce the airwayś pathogen burden (Safdar et al., 2009). A hypertonic seawater solution for nasal lavage enhanced barrier function increased the mucociliary clearance**,** and improved decongestion activity (Huang et al., 2019). Nebulized hypertonic saline has been reported to have a good safety profile and improves clinical severity scores and reduces the length of hospital stay in infants suffering from acute viral bronchiolitis (Zhang et al., 2017), a fact that was even recommended in the American Academy of Pediatrics guidelines (Baron and El-Chaar, 2016). Some reviews summarized the health-benefits after treatment with nebulized hypertonic saline for patients who have bronchiectasis and cystic fibrosis (Carro and Martinez-Garcia, 2019, Reeves et al., 2015).

Hypertonic salt solution augments bactericidal, virucidal, and anti-inflammatory activity (Munoz et al., 2018, Rubino et al., 2020, Michon et al., 2014) also in combination with Ibuprofen as a result of the amphipathic nature of hypertonic solutions. This was suggested as a promising preventing and therapeutic strategy to combat SARS-CoV-2 (García et al., 2020) and against Coxsackie-virus B3 to inhibit virus replication and, in further consequences, reducing heart tissue damage in viral myocarditis (Qiu et al., 2017). Antiviral-activity of high salt concentrations was further reported in both human and animal macrophages, thus strengthening the immune response when challenged with viruses (Zhang et al., 2018).

Filtering facepieces, which are used to protect their wearer unspecifically from infectious agents, do not inactivate infectious virus particles. If worn for a longer period of time, they might even act as concentrators of micro-organisms on their surfaces, and as a consequence, represent a certain risk of infection for their wearers. Impregnating such masks with salt could strongly reduce the numbers of infectious particles on their surfaces and thus protect mask carriers unspecifically but efficiently from infections. The focus of this line of experiments was quantification of possible protective effects of salt on the reduction of infectious agents.

## 2 Methods

Growth inhibition of hypertonic saline was evaluated on a robust unicellular organism with a defending cell wall i.e., in yeast (OSNA Nährmittel GmbH, Osnabrück, Germany). In a first step, a yeast suspension of 500 mg dried *Saccharomyces cerevisiae* in 50 ml *A. dest.* comprising 5% saccharose (Südzucker, Mannheim, Germany) was prepared. Microtitre wells were inoculated with 20 µl of this suspension. Thereafter 100 µl of 20% saccharose in *A. dest.* were added to each well followed by 100 µl of the appropriate double concentrated salt solutions i.e., 0, 10, 25, 50, and 100 (g/L) sodium chloride (NaCl) as well as a commercially available product (MIHESA®, Omnignostica Ltd., Höflein/Danube, Austria). The mixtures were kept in humid chambers at room temperature on a rocking device. Growth curves were determined by measuring the turbidity of each well at the beginning and after 24 h at 650 nm. All determinations were carried out in 10-fold replicates.

## 3 Results

**Fig. 1:**
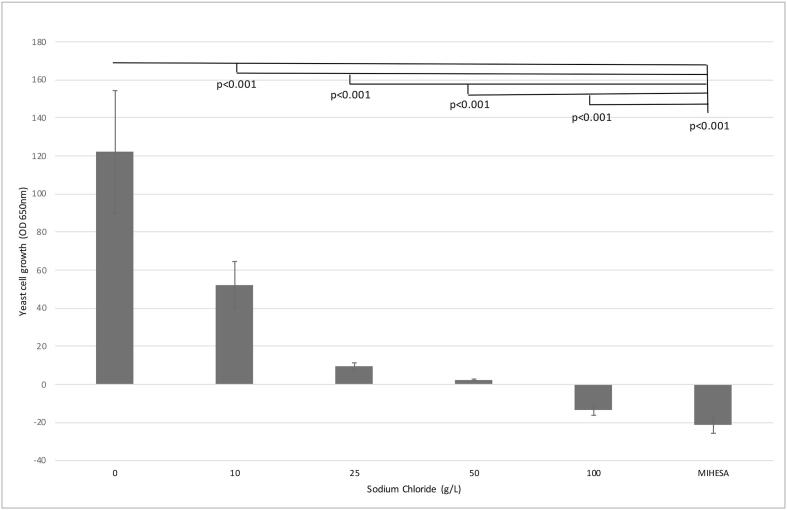
*Saccharomyces cerevisiae* was inoculated in an ascending sequence of sodium chloride solutions (10 to 100 g/L), including a commercially available product (MIHESA®). Growth inhibition upon increasing hypertonic saline was determined through turbidity measurement at 650 nm, and the delta between the two measurements was plotted as a percentage (±standard deviation) from the mean value of each 10 replications by the respective formula (OD2 – OD1/OD1*100). The two-sided T-test calculated significant differences.

After an incubation of 24 h, we observed a complete growth inhibition above 50 g/L NaCl. Above 100 g/L NaCl leads to decreased absorption units compared to the initial ones, thus indicating a decomposition of the original yeast suspension (Fig. 1).

## 4 Discussion:

These data affirm the hypothesis that salt crystal on the surface of the filtering face-piece might quantitatively inactivate infectious particles. If aerosols with infectious agents reaches the masḱs surface they become attracted by the hygroscopic salt crystal. Salt dissolves in the aerosol water droplets that proteins and nucleic acids lose their structural integrity and become inactivated concerning their infectious properties (Safdar et al., 2009). Such an unspecific effect of salt may not only protect mask surfaces from known infectious particles but even from new e.g. SARS-CoV-2 or mutated ones. As is generally known, sea air has health-promoting effects on respiratory diseases, among others, because the salt-rich air promotes blood circulation, making the mucous membranes more resistant to germs and infections. Moreover, salt-containing body fluids like sweat or tears were not noted as the main infection path (Xia et al., 2020) in contrast to the saliva (Han and Ivanovski, 2020). Filtering face-piece operate**s** through filtration without the ability to kill the viruses. If the filtration were combined with antiviral agents simultaneously in the masks, this would be much more efficient against cross-infection after being discarded (Zhou et al., 2020). We were awarded the utility patent (GM 50082/2020) for hypertonic saline coated filtering facepiece and allocated this idea to the personal application at onés own disposal. The preparation of a “hypertonic face mask” is simple and applicable to each filtering facepiece by spraying it with several pump strokes exterior and interior so that the whole mask will be covered with the salt solution. In case of hypersensitivity, the inner surface should not be sprayed completely. As was shown previously (Rubino et al., 2020) hypertonic filtering-face masks did not change the pressure drop significantly compared to bare membranes regardless of the salt concentration and membrane thickness. Also, in harsh conditions like a humid environment appear with a prolonged period of use, even an increased inactivation activity was reported in contrast to bare filters. This emphasizes salt-coated filters highly effective even after long-lasting breathing and ensuring reusability.

Besides the salty flavor of the filtering facepiece coated with a hypertonic salt solution**,** one should pay attention in hypertonic patients, and that high salt consumption might exacerbate autoimmune diseases (Scrivo et al., 2019). Nevertheless, overwhelming evidence exists for the antimicrobial effects and health benefits of hypertonic saline to support the immune system to defend against pathogens (Rucker et al., 2018). Besides the known antimicrobial action of hypochlorous acid, generated through myeloperoxidase, increased sodium chloride concentrations exert an antiviral activity as well (Ramalingam et al., 2018).

## 5 Conclusions

Hypertonic saline inhibited yeast cell proliferation in a dose-dependent manner. This effect was observed in a unicellular organism comprising a defending cell wall in contrast to viruses. Therefore, even speaking of COVID-19, this unspecific salt effect might not only enforce the protection of filtering facepiece but could even protect humans from this particle until an efficient vaccination is found for specific protection of individuals. An ongoing clinical trial (https://clinicaltrials.gov/ct2/show/NCT04465604) supports the currentness of hypertonic salt solutions in the battle against COVID-19. Even in the future, salt impregnated masks may protect from an upcoming pandemi**c**.

## CRediT authorship contribution statement

**Franz Tatzber:** Data curation, Investigation, Project administration, Supervision, Validation, Visualization, Writing - original draft, Writing - review & editing. **Ulrike Resch:** Data curation, Investigation, Project administration, Supervision, Validation, Writing - original draft, Writing - review & editing. **Meinrad Lindschinger:** Supervision, Validation, Writing - review & editing. **Gerhard Cvirn:** Supervision, Validation, Visualization, Writing - review & editing. **Willibald Wonisch:** Data curation, Investigation, Project administration, Supervision, Validation, Visualization, Writing - original draft, Writing - review & editing.

## Declaration of Competing Interest

The authors declare that they have no known competing financial interests or personal relationships that could have appeared to influence the work reported in this paper, except Wonisch W., who is affiliated with Omnignostica Ltd.
